# *ERBB3* is a marker of a ganglioneuroblastoma/ganglioneuroma-like expression profile in neuroblastic tumours

**DOI:** 10.1186/1476-4598-12-70

**Published:** 2013-07-08

**Authors:** Annica Wilzén, Cecilia Krona, Baldur Sveinbjörnsson, Erik Kristiansson, Daniel Dalevi, Ingrid Øra, Katleen De Preter, Raymond L Stallings, John Maris, Rogier Versteeg, Staffan Nilsson, Per Kogner, Frida Abel

**Affiliations:** 1 Department of Clinical Genetics, Institution of Biomedicine, Box 413, S- 405 30, Gothenburg University, Gothenburg, Sweden; 2 Childhood Cancer Research Unit, Karolinska Institute, Astrid Lindgren Children’s Hospital, Q6:05, S-171 76, Stockholm, Sweden; 3 Department of Medical Biology, University of Tromsø, Tromsø, Norway; 4 Department of Mathematical Statistics, Chalmers University of Technology, Gothenburg, Sweden; 5 Department of Pediatric Oncology, Clinical Sciences, Lund University, Lund, Sweden; 6 Center for Medical Genetics, Ghent University Hospital, Ghent, Belgium; 7 Department of Cancer Genetics, Royal College of Surgeons in Ireland and Children’s Research Centre, Our Lady’s Children’s Hospital, Dublin, Ireland; 8 Children's Hospital of Philadelphia, Division of Oncology, The University of Pennsylvania, Philadelphia, PA, USA; 9 Department of Oncogenomics, Academic Medical Center, Meibergdreef 9, 1105 AZ, Amsterdam, The Netherlands

**Keywords:** Microarray, Expression, Cancer, Systems biology, Oncology, Network, Reverse engineering, Unsupervised, Clustering, Cell cycle, Spindle assembly, Her-3, HER3, ERBB3, Her-2, HER2, ERBB2, EGFR, ERBB1, BIRC5, Survivin, MYCN, N-myc, ALK, PHOX2B, NTRK1, CCND1

## Abstract

**Background:**

Neuroblastoma (NB) tumours are commonly divided into three cytogenetic subgroups. However, by unsupervised principal components analysis of gene expression profiles we recently identified four distinct subgroups, r1-r4. In the current study we characterized these different subgroups in more detail, with a specific focus on the fourth divergent tumour subgroup (r4).

**Methods:**

Expression microarray data from four international studies corresponding to 148 neuroblastic tumour cases were subject to division into four expression subgroups using a previously described 6-gene signature. Differentially expressed genes between groups were identified using Significance Analysis of Microarray (SAM). Next, gene expression network modelling was performed to map signalling pathways and cellular processes representing each subgroup. Findings were validated at the protein level by immunohistochemistry and immunoblot analyses.

**Results:**

We identified several significantly up-regulated genes in the r4 subgroup of which the tyrosine kinase receptor *ERBB3* was most prominent (fold change: 132–240). By gene set enrichment analysis (GSEA) the constructed gene network of *ERBB3* (n = 38 network partners) was significantly enriched in the r4 subgroup in all four independent data sets. *ERBB3* was also positively correlated to the ErbB family members *EGFR* and *ERBB2* in all data sets, and a concurrent overexpression was seen in the r4 subgroup. Further studies of histopathology categories using a fifth data set of 110 neuroblastic tumours, showed a striking similarity between the expression profile of r4 to ganglioneuroblastoma (GNB) and ganglioneuroma (GN) tumours. In contrast, the NB histopathological subtype was dominated by mitotic regulating genes, characterizing unfavourable NB subgroups in particular. The high ErbB3 expression in GN tumour types was verified at the protein level, and showed mainly expression in the mature ganglion cells.

**Conclusions:**

Conclusively, this study demonstrates the importance of performing unsupervised clustering and subtype discovery of data sets prior to analyses to avoid a mixture of tumour subtypes, which may otherwise give distorted results and lead to incorrect conclusions. The current study identifies *ERBB3* as a clear-cut marker of a GNB/GN-like expression profile, and we suggest a 7-gene expression signature (including *ERBB3*) as a complement to histopathology analysis of neuroblastic tumours. Further studies of ErbB3 and other ErbB family members and their role in neuroblastic differentiation and pathogenesis are warranted.

## Background

Peripheral neuroblastic tumours (NT’s) are derived from developing neuronal cells of the sympathetic nervous system and are the most frequent extracranial solid tumours of childhood. NT’s are composed of variable proportion of neuroblasts (neuronal lineage) and Schwannian cells (glial lineage), and are classified into histopathological categories according to the presence or absence of Schwannian stromal development, differentiation grade of the neuroblasts, and their cellular turnover index. According to the International Neuroblastoma Pathology Classification (INPC - Shimada system) [1], the three subtype categories and their subtypes are: 1) Neuroblastoma (NB), Schwannian stroma-poor; 2) ganglioneuroblastoma (GNB), intermixed (Schwannian stroma-rich) or nodular (composite Schwannian stroma-rich/stroma-dominant and stroma-poor); 3) ganglioneuroma (GN), Schwannian stroma-dominant. Neuroblastoma exhibit an extreme clinical and biological heterogeneity, and patients are assigned to risk groups based on several criteria including stage [2,3], age [4], histological category and grade of tumour differentiation (histopathology) [5], the status of the *MYCN* oncogene [6], chromosome 11q status [7], and DNA ploidy [8] as the most highly statistically significant and clinically relevant factors [9]. One-half of NB patients have metastatic disease at diagnosis (INSS stage 4 or INRGSS stage M). All metastatic tumours with *MYCN* amplification (MNA) are aggressive and considered being high-risk tumours [9], whereas children with metastatic disease without MNA (approximately 65%) have variable clinical behaviours depending on age at diagnosis, histopathology, and other genetic factors. Based upon cytogenetic profiles, previous studies have categorized NB tumours into three major subtypes [10,11]: Subtype 1 representing favourable tumours with near triploidy and high expression of the Neurotrophic receptor TrkA (or NTRK1), mostly encompassing non-metastatic NB stages 1 and 2; subtype 2A representing unfavourable NB stages 3 and 4, with 11q deletion (Del11q) and 17q gain (Gain17q) but without MNA; subtype 2B representing unfavourable widespread NB stages 3 and 4 with MNA often together with 1p deletion (Del1p) and Gain17q. Several gene sets are shown to discriminate the molecular subgroups and risk groups by mRNA and microRNA expression profiling in neuroblastic tumours [12-21]. A recent expression analysis by our research group identified the three cytogenetically defined subtypes (1, 2A, and 2B) by unsupervised clustering, but further indicated the existence of a fourth divergent subgroup [12]. Moreover, we identified a 6-gene signature including *ALK*, *BIRC5*, *CCND1*, *MYCN*, *NTRK1*, and *PHOX2B* to successfully discriminate these four subgroups [12]. The fourth (r4) subgroup encompassed tumours characterized by Del11q and high expression of genes involved in the development of the nervous system, but showed low expression of *ALK*. Approximately 7-9% of sporadic NB cases show inherent *ALK* mutations [22,23], and *ALK* overexpression, both in its mutated and wild type form, is demonstrated to define a poor prognosis in NB patients [24]. In relation to this our previous findings suggests the Type 2A (r2) and Type 2B (r3) subgroups, which both display high *ALK* expression, to be driven by the ALK pathway. In contrast, the r4 subgroup displaying low expression of all six genes of the signature, is suggested to be driven by an alternative oncogenesis pathway.

In the present study we aimed to further investigate the expression profiles of the four subgroups, and r4 in particular. By differential expression analysis and reverse engineering we found *ERBB3* and its network members to be significantly overrepresented within the r4 tumour subgroup. Moreover, two other ErbB family members, *ERBB2* and *EGFR*, were found to show concurrently higher expression. In contrast, unfavourable neuroblastoma subgroups (r2 and r3) were specifically characterized by G2/M cell cycle transition and mitotic regulating genes. By expression analysis of histopathology categories (*i.e.* NBs, GNBs, and GNs) we found the r4 subgroup to show an identical expression profile to GNB/GN types, and overexpression of ErbB3 was also confirmed at the protein level in GN tumours. We conclude that the ERBB-profile (high expression of *EGFR*, *ERBB2* and *ERBB3*) defines a ganglion-rich neuroblastic tumour sub-set.

## Results

### Differential expression in r-subgroups

**Figure 1:**
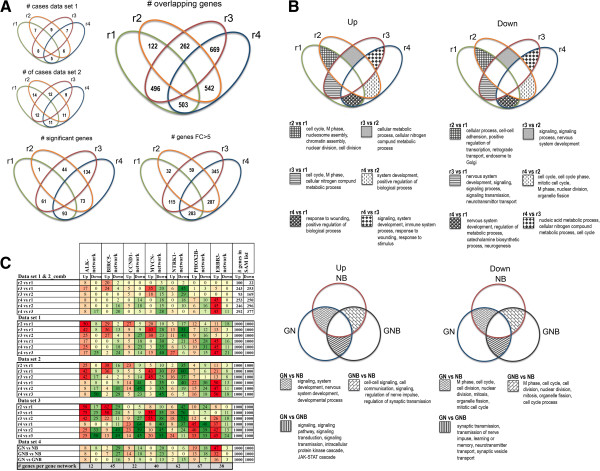
**Analyses of SAM gene lists. A**. Venn diagram of SAM results. SAM results from six r-group comparisons are presented for the DePreter and McArdle/Wilzén data sets. # cases = Total number of cases included in each comparison from each data set; # overlapping genes = Total number of genes overlapping between the original SAM gene lists (1000 genes from each direction) from data set 1 and 2, constituting the SAM_intersect_ gene lists; # significant genes = Total number of overlapping significant genes (combined p-value) using a cut-off of p < 6.25E-06, which correspond to a Bonferroni corrected p-value of p < 0.05; # genes FC > 5 = Total number of overlapping genes showing a combined fold change (FC) above 5 in each comparison. **B**. Venn diagram of GO functional themes. The Biological Networks Gene Ontology (BiNGO) tool in Cytoscape was utilized to map the predominant functional themes of the SAM gene lists. The most dominating Gene Ontology (GO) terms from each SAM comparison are presented from two differential expression directions; "up" (left panel) or "down" (right panel). Upper panel: r-group comparisons from all three data sets; data set 1 (DePreter), data set 2 (McArdle/Wilzén), and data set 3 (Wang). Lower panel: Histopathology group comparisons in data set 4 (Versteeg) The full GO search results are presented in Additional file 3. **C**. Gene network frequencies. The heat map table represents the percentage of network genes out of the total number of genes in the gene networks (marked in grey). The total number of genes in the SAM gene lists are presented to the right. The upper panel “Data sets 1 & 2_comb” represents combined intersect gene lists from the two data sets, and the other panels represents gene lists from the 1000 most differentially expressed genes in each direction.

To explore subgroup-specific characteristics we performed a differential expression analysis by SAM. Thirty-seven tumour cases from three studies were pre-processed in two separate data sets (data set 1, n = 14, and data set 2, n = 23, Table 1), and both data sets were divided into four r-subgroups based on rules according to the previously described 6-gene signature (6-GeneSig, Additional file 1) [12]. Six SAM pair-wise comparisons between r-subgroups were performed on each data set separately, and the 1000 most significant genes (according to descending SAM d-score) with a fold change above 2, were extracted to create SAM_intersect_ gene lists representing both data sets (Additional file 2). The r2 versus r1 group comparison showed 122 differentially expressed genes present in lists from both data sets, and the r3 versus r1 group comparison showed 496 overlapping genes (Figure 1A). The r4 subgroup showed the highest proportion of significant differentially expressed genes compared to all the other subgroups in both data sets (number of overlapping genes ranging between 503 and 669, Figure 1A).

**Table 1:** Data sets used in the current study

	**Name**	**Reference**	**# Total cases**	**Description**	**Analysis platform**	**# Analysis cases**	**Analysis groups**	**Purpose of study**
Data set 1	DePreter	[53]	17	Neuroblastoma	Affy HU133A (pre-amplified)	14	r-groups (r1-r4)	Differential mRNA expression of subgroups
Data set 2	McArdle	[54]	22	Neuroblastic	Affy HU133A	17	r-groups (r1-r4)	Differential mRNA expression of subgroups
	Wilzén	[55]	8	Neuroblastoma	Affy HU133A	6	r-groups (r1-r4)	Differential mRNA expression of subgroups
Data set 3	Wang	[56]	101	Neuroblastoma	Affy HGU95Av	67	r-groups (r1-r4)	Verification of subgroup findings; Gene network construction
Data set 4	Versteeg	[57]	110	Neuroblastic	Affy HU133plus2	110	Histology groups (GN, GNB, NB)	Differential mRNA expression of histology subgroups
Data set 5	Kogner	-	12*	Neuroblastic	IHC, WB	8 (IHC), 9 (WB)	Histology groups (GN, NB)	Protein expression validation in histology subgroups

*SAM* Significance Analysis of Microarray. Neuroblastic = mixed pool of neuroblastoma (NB), ganglioneuroblastoma (GNB), and ganglioneuroma (GN). *Affy* Affymetrix, *IHC* Immunohistochemistry, *WB* Western blotting.* One case overlaps with data set 2 (case "NBS1" in data set 2 = case ′6′ in data set 5).

The r1 subgroup (corresponding to the cytogenetically defined subgroup Type 1) was found to mainly involve nervous system developmental and catecholamine metabolic process related genes. In the MNA-specific subgroup r3 (corresponding to Type 2B), *KIF15* was the most significantly up-regulated gene (fold = 15) while *CUX2* showed the highest expression fold change (fold = 17). The *MYCN* gene was found on the 74^th^ position of up-regulated genes (fold = 9), and *NTRK1* was identified as the most significantly down-regulated gene within r3 compared to r1 (fold = 80, Additional file 2). Also, *LMO3* and *PHGDH* were found to be specifically up-regulated in the r3 subgroup compared to the other subgroups. High expression of *ALK* was found in both the r2 (2-fold) and r3 (5-fold) subgroups compared to the favourable r1 subgroup. Moreover, r2 and r3 also showed up-regulation of several G2/M cell cycle transition and mitotic checkpoint related genes (*e.g. AURKA*, *BRCA1*, *BUB1B*, *CCNA2*, *CCNB1, KIF15, MCM2, MCM3*, and *MCM5 etc.*), which in contrast showed a significant down-regulation in the r4 subgroup. In line with this, a Gene Ontology (GO) search identified “cell cycle” as the most significant process accumulated in the SAM_intersect_ gene lists of the r2 and r3 subgroups (Figure 1B, Additional file 3). The apparent overrepresentation of cell cycle-related genes in subgroups r2 and r3 encouraged us to investigate enrichment of other cell cycle key players and networks in our SAM gene lists.

### Differential expression in subgroup r4

Among the 10 most significantly up-regulated genes in the r4 subgroup in data sets 1 and 2, the following eleven genes were found; *ABCA8, APOD, ASPA, CDH19, ERBB3, FXYD1, ITIH5, MAL, PLP1, S100B,* and *ST6GALNAC2*. According to the GO search, these genes are mainly involved in nervous system development, multicellular organismal development, and response to wounding (Figure 1B, Additional file 3). *ERBB3* was found as the “top-one” up-regulated gene in r4 versus r3 with a 240-fold expression. *ERBB3* encodes a transmembrane tyrosine kinase receptor, which has previously been associated with cancer in a large number of studies (>500 publications). ErbB3 is activated through dimerization to one of its four structurally related family members; EGFR, ErbB2, or ErbB4. ErbB-family members are often co-expressed, and thus we found it relevant to investigate their expression level relationships in our four neuroblatic data sets. We found a positive significant correlation of *ERBB3* to the *EGFR* and *ERBB2* family members, and a negative correlation to all genes of the 6-GeneSig in all four data sets (p < 0.05, Additional file 4). Also, *EGFR* and *ERBB2* showed a significant up-regulation in r4 subgroups of most data sets (p < 0.05, Additional file 2). *ERBB3* show several similarities to *ALK*, encoding the NB familial gene [25], and thus made a good candidate gene with potential role in the tumour development of r4 tumour types.

Among the down-regulated genes in the r4 subgroup *CACNA2D3* was the most significant in comparison to the r1 subgroup (50-fold change). This gene was also found to be the 25^th^ most down-regulated gene in the r3 subgroup compared to r1 (Additional file 2). Since both the r3 and r4 subgroups are previously found to show unfavourable outcome and poor survival [12], and the *CACNA2D3* gene is located in the 3p21.1- locus commonly deleted in many NB tumours, this encouraged us to further screen the SAM_intersect_ gene lists for other conceivable and previously reported tumour suppressor (TS) candidate genes. Out of 33 previously reported TS candidate genes, 15 were present among the SAM_intersect_ gene lists from data sets 1 and 2 (Additional file 5).

### Gene network construction and gene set enrichment analysis (GSEA)

**Figure 2:**
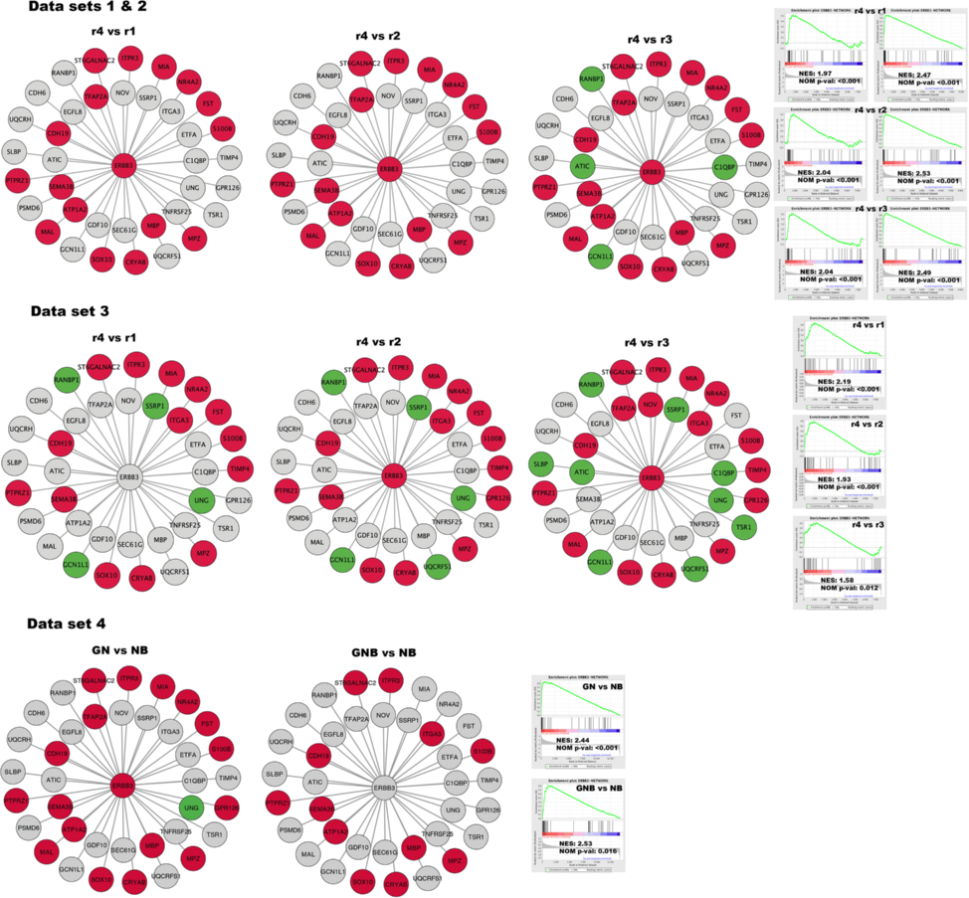
**Analyses of the ERBB3 gene network.** Gene network of ERBB3 (n = 38) for all data sets showing differentially expressed genes of r-groups (data sets 1–3) and histopathology groups (data set 4). Nodes are coloured as follows: Red = Up-regulated, Green = Down-regulated, Grey = Not affected. **Upper panel:** Data sets 1 and 2 (DePreter and McArdle/Wilzén) presenting three r-group comparisons (r4 vs. r1, r4 vs. r2, and r4 vs. r3). Only genes that were common in both data set 1 and 2 with fold change > 2 were included (*i.e.* SAM_intersect_ gene lists). **Middle panel:** Data set 3 (Wang) presenting three r-group comparisons (r4 vs. r1, r4 vs. r2, and r4 vs. r3). Genes included were those present in SAM gene list representing the 1000 most differentially expressed (ranked after significance). **Lower panel:** Data set 4 (Versteeg) presenting two histopathology group comparisons (GNB vs. NB and GN vs. NB). Genes included were those present in SAM gene list representing the 1000 most differentially expressed (ranked after significance). Gene set enrichment analysis (GSEA) plots of the ERBB3 network are according to gene list sorting mode = real, sorted in descending order. NES = Normalized enrichment score, NOM p-val. = Nominal p-value, according to the GSEA results (see Additional file 7).

Network modelling reveals the regulatory relationships among genes and can provide a systematic understanding of molecular mechanisms underlying biological processes. A variety of algorithms have been developed, and in the current study we chose the ARACNE algorithm [26] for reconstruction of seven networks (*ALK*, *BIRC5*, *CCND1*, *ERBB3*, *MYCN*, *NTRK1*, *PHOX2B*) from the Wang data set (n = 102), since this method has a documented high performance [27]. Also, 4850 pre-existing curate gene sets (c2) from the Molecular Signatures Database (MSigDB) were selected (Additional file 6). We subsequently analysed the lists of differential expressed genes for enrichment of these 4857 gene networks. The SAM_intersect_ lists of genes up-regulated in the r4 group were found to comprise 17 out of 38 partners (~ 45%) of the ARACNE_ERBB3 network (Figure 1C), which was significantly verified by GSEA (p < 0.001, Figure 2, Additional file 7). A relatively large fraction (between 20% and 58%) of the ARACNE_BIRC5 network partners (n = 45, Additional file 6) were found among the up-regulated genes of r2 and r3 tumour subgroups, which was also significant by GSEA (p < 0.001, Additional file 7). A GO search of the BIRC5 network partners suggested a role in mitosis (GO terms: cell cycle, nucleosome assembly, chromatin assembly, protein-DNA complex assembly, nucleosome organization, mitotic cell cycle, cell cycle phase, DNA packaging, M phase, and cell cycle process, data not shown). Other cell-cycle or mitotic related gene sets found to be enriched among the r2 and r3 subgroups were *e.g.* ZHOU_CELL_CYCLE_GENES_IN_IR_RESPONSE_24HR,WHITFIELD_CELL_CYCLE_LITERATURE, REACTOME_CELL_CYCLE_MITOTIC, REACTOME_CELL_CYCLE_CHECKPOINTS curate gene sets (Additional file 7).

### Verification of gene network modelling and differential expression analysis

The differential expression profiles of the r-subgroups were verified by replicating the study using the Wang data set (n = 67 cases, Table 1). The outcomes of SAM were in consistence with previous findings, showing the *ERBB3* gene to be significantly up-regulated and its gene-network partners to be significantly overrepresented in the r4 subgroup (Figure 1C, Figure 2). Also, several other previously identified r4-specific genes, *APOD, CDH19, FXYD1*, and *S100B,* were found among the 1000 most significantly up-regulated genes. In concordance with the previous analysis, we found the expression of *CUX2* (fold = 5), *LMO3* (fold = 2.7) and *PHGDH* (fold = 1.9) to be significantly higher in the MNA subgroup (r3) compared to the favourable subset (r1). In addition, cell cycle-related genes dominated the r2 and r3 subgroups, and this was significantly proven by GSEA of the BIRC5 network and other cell cycle networks (p < 0.001, Additional file 7).

To confirm the robustness of the ARACNE constructed gene networks, we selected the r3 versus r1 comparisons in data sets 1 and 2 to investigate the expected overrepresentation of MYCN- and NTRK1-network partners. Fourteen genes out of 40 (35%) of the ARACNE_MYCN network were found in the up-regulated gene lists, while eight out of 40 (20%) genes were found in the down-regulated gene lists, demonstrating an accumulation of the ARACNE_MYCN network in the r3 subgroup (Figure 1C, Additional files 7 and 8). Also, an accumulation of the ARACNE_NTRK1 network was found in the opposite direction. Out of 62 genes composing the ARACNE_NTRK1 network, 28 genes (~ 45%) were among the 1000 most down-regulated genes in r3, which was significant by GSEA (Additional files 7 and 8). According to significance by SAM, the *NTRK1* gene was the “top-one” down-regulated gene within the r3 versus r1 subgroup comparison in both data sets (fold change: >70, Figure 1C, Additional file 2). From these facts we conclude our study design to be substantial, and the constructed gene networks by ARACNE to be reliable and highly representative.

In addition, we checked the enrichment of network partners to the 6-GeneSig (*ALK*, *BIRC5*, *CCND1*, *MYCN*, *NTRK1*, and *PHOX2B*) and found the network representations to be in concordance with the 6-GeneSig expression levels in r-subgroups (Additional file 7). The credibility of ARACNE constructed networks were also tested by literature verification, and seven out of 38 transcriptional connections of the ERBB3-network as well as 11 out of 40 transcriptional connections of the MYCN-network were verified to have a functional relationship (data not shown). This demonstrates the robustness of the computationally inferred network analysis.

### Differential expression analysis of histopathology groups (data set 4)

To further explore the *ERBB3* expression among other neuroblastic tumour we utilized the R2 database (http://hgserver1.amc.nl), and found indications of high *ERBB3* expression in GNB and GN tumours. To investigate this finding in more detail, we performed a differential expression analysis of the histopathology subtypes in the Versteeg 110 data set (n = 110, Table 1). As expected, the *ERBB3* gene and networks partners were significantly enriched in GNB and GN tumours compared to NB (Figure 1C, Additional file 7). The highest enrichment of the ERBB3-network was found in GN tumours, with 18 up-regulated genes out of 38 (p < 0.001, Figure 2). In contrast, cell cycle-related genes and gene networks significantly dominated the NB types, including the ARACNE_BIRC5 network (Additional files 2 and 7).

### Subgroup-specific expression profiles

**Figure 3:**
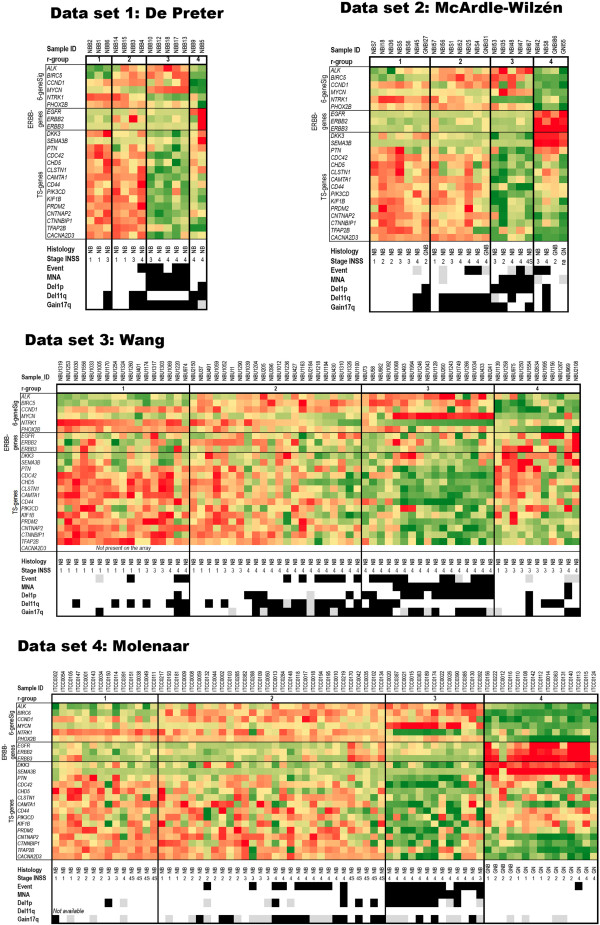
**Heat maps of r-subgroups.** Heat map of the 6-GeneSig (*ALK*, *BIRC5*, *CCND1*, *MYCN*, *NTRK1*, and *PHOX2B*), ERBB-genes (*EGFR*, *ERBB2*, and *ERBB3*) and 15 TS-genes (Tumour suppressor candidate genes) for all four data sets. The heat map colour scale is based on standard deviations (sd) of all cases in each data set separately, and ranges from sd = 2 (red) to sd = -2 (green). The lower panel represents clinical and biological markers: Histopathology, NB = Neuroblastoma, GNB = ganglioneuroblastoma, GN = ganglioneuroma; Stage INSS, clinical stages 1–4, and 4S according to the international staging system, na = not available; Event = dead of disease, MNA = *MYCN* amplification, Del1p = 1p deletion, Del11q = 11q deletion, Gain17q = gain of 17q. Colour coding: white = no event, black = event, grey = not available.

ErbB family member genes (*ERBB*-genes; *EGFR*, *ERBB2*, and *ERBB3*) and 15 previously reported tumour suppressor candidate genes (TS-genes) were next studied by heat maps in all four data sets (Figure 3). Most TS candidate genes were down-regulated in the MNA-specific r3 subgroup only. However, the *CTNNBIP1* and *KIF1B* transcripts were also found to be down-regulated in both r3 and r4 subgroups, and the *TFAP2B* transcript was specifically down-regulated in the r4 subgroup alone (Figure 3, Additional file 2). Overall, the expression profiles of the 6-GeneSig genes, *ERBB*-genes, and TS-genes (25 genes in total) among r-subgroups were very similar between data sets. Moreover, the expression profiles of the GNB/GN tumours were identical to the previously detected r4 subgroups of NB (Figure 3). These results strongly indicate that the same cellular pathways are active in r4 and GNB/GN tumours types, hence the ERBB-gene profile most likely represents a more differentiated subset of tumours.

### Verification of ErbB3 at protein level (data set 5)

**Figure 4:**
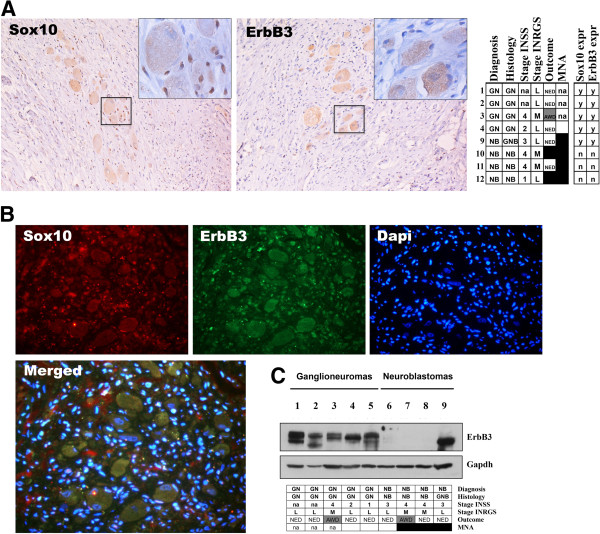
**Protein expression validation of ErbB3. A**. Immunohistochemistry (IHC) assessment of case 2 showing ErbB3 expression in the mature ganglion cells, and Sox10 expression in both ganglion and schwannian cells. Tumour tissue sections were stained using horseradish peroxidase (HRP) secondary antibodies. The right panel shows the Sox10 and ErbB3 expression results from all IHC assessments performed, y = yes, n = no expression. **B**. Colocalization study of ErbB3 and Sox10, showing ErbB3 expression mainly in ganglion cells. Tumour tissue sections were simultaneously fluorescently stained using anti-goat Alexa Fluor 594 (Sox10, red) and anti-mouse Alexa Fluor 488 (ErbB3, green), and Dapi (DNA, blue). **C**. High protein levels in GN tumours shown by Western blot of ErbB3. Primary antibodies used in studies: Sox10 ([N-20], Santa Cruz Biotechnology), and ErbB-3 ([RTJ2], Abcam), which binds to both phosphorylated and unphosphorylated forms. Case 6 was also included in the microarray analysis (NBS1, data set 2). Clinical data: NB = neuroblastoma, GNB = ganglioneuroblastoma, GN = ganglioneuroma; INSS stage [2]; INRGSS stage [3]: L = Localised, L1/L2 (INSS 1, 2, 3); M = Metastatic (INSS 4); Outcome: NED = No evidence of disease (white); AWD = Alive with disease (grey); DOD = Dead of disease (black); MNA (MYCN-amplification), black = yes, white = no, na = not applicable.

To validate the biological significance of the *ERBB3* enrichment in the expression profiles of GN tumours, the ErbB3 protein expression was investigated by immunohistochemistry (IHC) and western blot (WB) analysis. The IHC was performed on formalin-fixed and paraffin-embedded (FFPE) tissue slides from four GN and four NB tumours by using antibodies specific for Sox10 ([N-20], Santa Cruz Biotechnology) and ErbB-3 ([RTJ2], Abcam) respectively. The IHC showed ErbB3 to be mainly expressed in mature ganglion cells, whereas Sox10 was expressed in both ganglion and schwannian cells (Figure 4A-B). A high fraction of satellite cells, as well as schwannian cells were also Sox10 immunopositive (data not shown).

Immunoblot analysis was performed on five GN and four NB in total (data set 5, Table 1). Out of the five investigated GN cases, four corresponded to the GN cases examined by IHC. In addition, the WB analysis also included one NB encompassed in the microarray analysis (case 6 corresponding to NBS1 in data set 2). The same antibody as for IHC ([RTJ2], Abcam) directed against the cytoplasmic region of ErbB3 was chosen in order to detect several isoforms of the protein as well as post-translationally modified and unmodified forms. Overall, ErbB3 expression levels were high and clearly enriched in the GN subset compared to the NB subset, which showed no detectable levels of ErbB3. Moreover, case 6/NBS1 previously displaying no or very low expression of *ERBB3* by microarray analysis (data set 2, Figure 3), showed no detectable levels of ErbB3 at protein level by immunoblot analysis. Only one of the NB tumours (case 9) showed a strong ErbB3 signal. However, this case was a localized INSS stage 3 with favourable biology, later histopathologically classified as a GNB. Moreover, only the lower molecular weight band was visible indicating that the protein might be in its inactive unphosphorylated form, or indicate other post-translational modification or isoforms of ErbB3 (Figure 4C).

### Histopathology classification

Based on our results we included *ERBB3* in the 6-GeneSig thus creating a new 7-GeneSig. The 7-GeneSig was refined to discriminate five subclasses; “NB-r1”, “NB-r2”, “NB-r3”, “GNB-r4”, and “GN-r4” (Additional file 9). In order to test the robustness of this 7-GeneSig subgroup classification, cases from all data sets were reclassified into three histopathology prediction classes “NB” (NB-r1, NB-r2, NB-r3), “GNB” (GNB-r4), and “GN” (GN-r4) and the reliability of assignments were investigated. Out of 110 neuroblastic tumours of the Versteeg data set, 82 cases could be successfully assigned according to the 7-GeneSig rules (Additional file 9). All NB histopathology types (64 out of 64) were correctly assigned according to the 7-GeneSig, and the inter-rate reliability of assignments was highly significant (Kappa measure of agreement p = 7.489E-17, Table 2). Five out of eight GNB tumour types, as well as nine out of ten GN tumour types were correctly assigned. One GN was predicted as “GNB” according to the 7-GeneSig (Table 2). In addition, we also performed a reassignment test on data set 2 comprising one GN, four GNB, and 25 NB tumour types, which was also significant (inter-rate reliability p = 0.003, data not shown). Reassignment of r4-cases (from data sets 1, 2 and 3), previously classified as NB, were all assigned to the “GNB” or “GN” categories by the 7-GeneSig. Also, all NB cases of data set 4 fell into the NB r1-r3 categories (data not shown). Conclusively, the histopathology classification and subgroup assignment by the 7-GeneSig seemed reliable and highly predictive.

**Table 2:** Histology prediction by 7-GeneSig

		**Histology**				
		NB	GNB	GN	Total:	
**Predicted**	NB	64	0	0	64	
	GN	0	1	9	10	
	GNB	2	5	1	8	
	Total:	66	6	10	**82**	*nd=28*
*Measure of agreement (Kappa) p= 7.489E-17*						

Histology prediction was performed on the Versteeg data set (n = 110). Group prediction was based upon the standard deviation (sd) of expression of *ALK*, *BIRC5*, *CCND1*, *MYCN*, *NTRK1*, *PHOX2B*, and *ERBB3* according to the rules in Additional file 9. Out of 110 tumours, 82 were successfully assigned and for 28 tumour cases no group belonging could be determined. The groups were assigned as follows: "NB" (r1-r3),"GNB" (r4-GNB), and "GN" (r4-GN). The inter-rate reliability (Kappa) was used to measure the agreement between the assignments of categories (p= 7.489E-17).

## Discussion

Neuroblastic tumours (NT’s) represent a spectrum of disease, from undifferentiated and aggressive NB to the differentiated and largely quiescent GN tumours. NB tumours are commonly categorized into three main types based on numerical and structural genomic alterations, as well as expression of the neurotrophin receptor TrkA [10]. In a recent study using Principal Components Analysis (PCA) however, our data indicated the existence of four molecular tumour groups, r1-r4 [12]. In the current study we aimed to further characterize these four molecular subgroups, and investigated the divergent r4 group in particular. While the r2 (Type 2A) and r3 (Type 2B) tumour subgroups were dominated by cell cycle-related genes and networks, those were completely absent in the r4 subgroups (data sets 1–3) and GNB or GN subtypes (data set 4). The vast majority of the cell cycle-related genes were linked to the G2/M transition and spindle assembly checkpoint (*e.g. BIRC5*, *BRCA1*, *BUB1B*, *CCNA2*, *CCNB1*, *FANCI*, *HMMR*, *KIF15*, and *MCM2*), many of which were found to belong to the ARACNE-modelled BIRC5-network. Overexpression of genes involved in mitotic regulation is typical for rapidly proliferating tumours and would also be expected to be enriched in the aggressive NB subtypes when compared to more differentiated quiescent GNB and GN tumours. The BIRC5 protein is found to stabilize the microtubules in the chromosomal passenger complex, and knockdown of *BIRC5* causes apoptosis in NB via mitotic catastrophe [28]. Also, a previous publication show that NB tumours with genomic aberrations in G1-regulating genes leads to S and G2/M phase progression [20]. Interestingly, the fork head box (FOX) gene *FOXM1* encoding a protein phosphorylated in M phase was significantly up-regulated in r2 and r3 subgroups. FOXM1 activates the expression of several cell cycle genes, *e.g. AURKB*, *CCNB1*, *CCND1*, *MYC*, and is involved in cell proliferation and malignancy [29]. Several cell cycle and DNA repair genes, including *BIRC5*, are suggested to act downstream of N-myc [21,30,31]. In addition, most of the studied tumour suppressor (TS) candidates were specifically down-regulated in the r3 subgroup, which is probably explained by them acting downstream of N-myc. Several of the TS candidate genes are also located in the 1p36 chromosomal region (*e.g. CHD5* and *KIF1B*[32-34]), and Del1p is a well-known prognostic marker highly correlated to *MYCN*-amplification in NB [35]. One such N-myc-regulated and 1p36-localized TS candidate is *CDC42*, encoding a small GTPase protein. This protein have a function in cell polarization and growth cone development in NB cell differentiation, similar to Rac1 and Cux-2, and is suggested to inhibit neuritogenesis in NB [36]. In concordance to this, we found *CDC42* to be the 14^th^ most significantly down-regulated gene in the MNA subgroup (r3) compared to subgroup r2.

The main focus of the study was to define the underlying regulatory networks of the r4 subgroup. In contrast to the other three well-known subgroups of NB, the r4 tumours showed high expression of embryonic development and nervous system signalling genes. One of the most prominent genes from the differential expression analysis was *ERBB3*, encoding a member of the epidermal growth factor receptor (EGFR) family of receptor tyrosine kinases (RTK’s). The ARACNE-modelled ERBB3-network was significantly enriched in the differentially expressed gene lists of the r4 subgroups (data sets 1-3), and this enrichment was also found in the GNB and GN histopathology categories of data set 4. Two members of the ERBB3-network, *S100B* and *SOX10*, were among the ten most significantly up-regulated genes in the r4 subgroups. The S100 calcium binding protein B (S100B) has long been reported as a prognostic biomarker of malignant melanoma [37], and a paired down-regulation of *ERBB3* and *S100B* is observed in malignant peripheral nerve sheath tumours confirming their functional relationship [38]. Interestingly, the S100 beta protein, mapping to chromosome 21, has been proposed to be responsible for the lack of NB in Down syndrome patients by producing growth inhibition and differentiation of neural cells [39]. The SRY box 10 transcription factor (Sox10) is a key regulator of the developing nervous system, and has been shown to control expression of ErbB3 in neural crest cells [40,41]. A paired overexpression of ErbB3 and Sox10 has been observed in pilocytic astrocytoma (PA) a common glioma of childhood, which verifies their network connection found in the current study [42]. Also, Sox10 and S100 are routinely employed in the pathological diagnosis of neural crest-derived tumours [43], and Sox10 serves as an embryonic glial-lineage marker in NT’s [44]. By immunohistochemistry assessment, we found Sox10 to be expressed in both the schwannian cells and ganglion cells, whereas ErbB3 was found mainly in the mature ganglion cells. We could also verify the GN-specific expression of ErbB3 by immunoblot analysis.

ErbB3 is activated through ligand binding of neuregulin (NRG), leading to heterodimerization of ErbB3 to other ErbB members and subsequent phosphorylation. Activated ErbB3 regulates proliferation through downstream signalling of the phosphoinositol 3-kinase/AKT survival/mitogenic pathways [25]. In the current study we found a significant correlation of *ERBB3* to its family members *EGFR* and *ERBB2* in all four independent data sets. *EGFR* and *ERBB2* were also both found to be significantly up-regulated in all r4 subgroups as well as in the GNB and GN tumours. Amplification of *ERBB3* and/or overexpression of its protein has been reported in numerous cancers, including prostate, bladder, and breast. Moreover, loss of ErbB3 function has been shown to eliminate the transforming capability of ErbB2 (also known as HER-2) in breast tumours [45]. Although the extent of the role of ErbB3 is emerging, its clinical relevance in different tumours is controversial. There are a few studies of ErbB/HER receptor expression in neuroblastoma, showing that ErbB/HER family members in neuroblastic tumour biology is interrelated and complex, but their expression level may present a prognostic factor for patients outcome [46-48].

The heat map of 25 genes including the 6-GeneSig genes, *ERBB*-genes and TS-genes showed a very specific expression pattern among the different r-subgroups and histopathology categories. The similarity of expression profiles between the four data sets was striking. The correspondence of the r4 subgroups to the GNB and GN histopathology subtypes was obvious, and *ERBB3* appeared as a clear-cut marker for a GNB/GN-like expression profile. To demonstrate this further, a new 7-GeneSig (including *ERBB3*) was constructed and used in a histopathology reassignment classification test. The 7-GeneSig successfully assigned 100% NB tumours, 62,5% GNB tumours, and 90% GN tumours into the correct histopathology category (Kappa measure of agreement p = 7.489E-17, data set 4). Also, all r4-tumour types from data sets 1–3 were categorized as GNB or GN tumours according to the 7-GeneSig. By these facts we conclude that the NB tumours previously assigned to the r4 subgroup by the 6-GeneSig, most likely represent more differentiated NT’s and are seemingly GNB/GN tumours types. Our study brings out the complexity in classifying neuroblastic tumours. The previously described unfavourable characteristics and poor outcome of the r4 tumour group is puzzling [12], but can be explained by the fact that prognostic subsets of GNB’s exist [49]. Historically, GNB’s have been the most difficult of the NT’s to define in a consistent and uniform fashion, because the number and degree of differentiation of the neuroblastic cells tend to vary between cases as well as between different microscopic fields in the same tumour [1]. Moreover, the data sets used in the current study are probably not truly population-based, and the r4 subgroups found probably consist of different proportions of F/UF subsets. In addition, some tumours may previously have been misclassified as NB, or the tumour tissue part analysed by microarray may not be the same as the tissue part that underwent histopathology assessment. Furthermore, it is not clear whether differentiation markers are superior to other prognostic factors in defining outcome. Unfavourable markers such as MNA and clinical stage may also be present in or among differentiated cells, and mark a poor prognosis by themselves.

ErbB3 also has an important role in differentiation of Neural crest cell (NCC) lineages during the embryonic development [50]. Although ErbB receptors are also found to mediate proliferation and survival [47,48], the ERBB-profile found in this study is likely to reflect the phenotype or differentiation stage of developing neuronal progenitors. Upon induction of differentiation, neuronal progenitors may follow a variety of stages of NCC lineages. For example, neuroblasts in culture are shown to represent an immature bilineage stage able to progress towards neuronal and glial fates [44]. Schwannian cells are the principal glia of the peripheral nervous system, whereas neuroblasts differentiate from neural stem cells and exhibit variable degrees of differentiation up to ganglion cells. In this context, the ERBB-profile seems to be a marker of ganglionic-neuronal differentiation. A recent immunohistochemistry study of ErbB2 in neuroblastic tumours supports this conclusion [51]. However, it still remains uncertain whether the r4 subgroup of datasets 1 and 3 are indeed GN or GNB, or if the ERBB expression profile just marks the gradually differentiated NB tumours (encompassing increased levels of mature ganglion cells). Nevertheless, the results from all data sets are consistent in regards to the expression profile of the 25 genes selected for the heat map, strengthening the robustness of the suggested 7-gene signature. Accordingly, we propose ErbB3 as an excellent marker of neuronal differentiation, and suggest mRNA expression profiling by the 7-gene signature as a complement to histopathological assessment. However, the exact cut-off expression levels for classification needs to be worked out in more detail, and classification must be based on international standard cases and assays.

## Conclusions

In summary, by differential expression analysis and network modelling we have identified genes and gene networks constituting molecular and histological subgroups of neuroblastic tumours. The primary aim of our study was to identify genes characterizing the previously unknown r4 subgroup. Our results pinpointed *ERBB3* and its network as one of the most significantly up-regulated genes within this group. By studying the expression profiles in a broader range of neuroblastic tumour types, we found the r4 subgroup to be highly similar to GNB/GN tumour types. The ERBB-dominating profile found in r4 and GNB/GN tumours was clearly divergent from the cell-cycle-dominating profile mainly representing NB tumour subgroups (specifically unfavourable NB subgroups). Our findings indicate that the previously identified r4 subgroup most likely constitutes GNB/GN tumours or NB tumours with high content of mature ganglion cells. This study also demonstrates the importance of performing unsupervised subtype clustering prior to down-stream analyses. Predefined subgroups and supervised clustering studies might give distorted results if they are based on pools of mixed tumour histopathology subgroups. In conclusion, we have identified *ERBB3* as a marker of a GNB/GN-like expression profile, and we suggest a 7-gene expression signature as a complement to histopathological assessment of neuroblastic tumours. Further studies of ErbB3 and other members of the ErbB family and their role in neuroblastic differentiation and pathogenesis are warranted.

## Methods

### Pre-processing microarray data

Data from five published neuroblastoma expression microarray studies run on three different Affymetrix platforms (HU133A, HGU95Av, and HU133plus2) were used in this study (Table 1). Raw data files were obtained from Array Express (http://www.ebi.ac.uk/microarray-as/ae/) and Gene Expression Omnibus (http://www.ncbi.nlm.nih.gov/geo/), or directly from collaborators. Expression data files were normalized by gcRMA using Bioconducter (library BioC 2.4) in R 2.9.2 [52] in four separate groups; 1) the De Preter [53] data set run on the HGU133A Affymetrix platform (17 samples, preamplified), 2) the McArdle [54] and the Wilzén [55] data sets run on the HGU133A Affymetrix platform (30 samples, not pre-amplified), 3) the Wang [56] data set run on the HGU95Av2 platform (102 samples, not pre-amplified), and 4) the Versteeg [57] data set run on the HU133plus2 platform (110 samples). For each probe-set, the maximum expression values over all samples were determined, and probe-sets which showed very low or no detectable expression levels were filtered out (log2 expression <5). Next, the mean log2 expression level for each Gene symbol was calculated to generate “mean-per-gene” data files: 7439 genes in data set 1, 8106 genes in data set 2, 7542 genes in data set 3, and 15614 genes in data set 4.

### Differential expression analysis

NB samples from the DePreter and McArdle/Wilzén data sets were divided into four r-subgroups by a 6-gene signature (further referred to as the“6-GeneSig”) according to Abel et al., 2011 [12] (Additional file 1). From these two data sets, 14 (preamplified, De Preter) and 23 (non-preamplified, McArdle/Wilzén) cases respectively were successfully assigned into one of the four r-groups (Table 1). Differential gene expression analysis was performed by a two group unpaired Significance Analysis of Microarray (SAM) test (*i.e.* six comparisons) [58]. Gene lists comprising the 1000 most significantly differentially expressed genes (sorted after the d-statistic) with a fold change above 2 were exported from each comparison, from each direction (up or down), and from each data set, separately (resulting in 12 SAM gene lists per data set). Next, SAM gene lists from the two different data sets were compared, and 12 intersection gene lists (SAM_intersect_) were created. Too minimize the variance, a combined fold change (FC_comb_) for each gene in the SAM_intersect_ gene list was calculated as follows:

$$FC_{\mathrm{comb}}=\mathrm{FC}\frac{V_{2}}{1}\mathrm{FC}\frac{V_{1}}{2}$$

where FC_*i*_ is the fold change in data set *i* and

$$V_{i}=\frac{\mathrm{SE}\frac{2}{i}}{\mathrm{SE}\frac{2}{1}+\mathrm{SE}\frac{2}{2}}$$

where SE_*i*_ is the standard error of the mean log2 expression values in data

set *i*.

A combined p-value (P_comb_) for each gene in the SAM_intersect_ gene list was calculated as follows:

$$P_{\mathrm{comb}}=\Phi \left(\sqrt{\frac{N_{1}}{N_{1}+N_{2}}}\Phi^{-1}\left(P_{1}\right)+\sqrt{\frac{N_{1}}{N_{1}+N_{2}}}\Phi^{-1}\left(P_{2}\right)\right)$$

where N_*i*_ is the total number of samples of the two groups compared by the

d-statistic in SAM, and P_*i*_ the corresponding p-value for dataset *i*. Φ is the cumulative distribution function of the standard normal distribution and Φ^-1^ is its inverse function.

Based on an approximation of 8000 multiple tests (*i.e.* 8000 genes), a nominal p-value <6.25E-06 was found to correspond to an adjusted p-value <0.05 (according to Bonferroni correction) and was subsequently used as a cut-off for significance in SAM.

### Gene network modelling

A large gene regulatory network was constructed from an independent data set (Wang) of 102 expression profiles [56]. Mutual information values were estimated with the ARACNE (Algorithm for the Reconstruction of Accurate Cellular Networks) algorithm using a p-value cut-off of 1E-7 [26]. The data processing inequality (DPI) was applied with a tolerance of 0.15. Gene networks of seven selected genes were extracted from the global network together with their immediate gene neighbours. The gene networks of nearest neighbours were visualized in Cytoscape 2.8.2.

### Gene ontology (GO) and Gene Set enrichment analysis (GSEA)

Ranked SAM gene lists (by d-statistic) from the separate data sets were investigated for Gene Ontology terms using BiNGO 2.4 (Biological Network Gene Ontology, http://www.psb.ugent.be/cbd/papers/BiNGO/). The Gene Set Enrichment Analysis (GSEA, http://www.broad.mit.edu/gsea/) software was used to investigate whether a gene network was significantly overrepresented in the different r-subgroups. The enrichment tests were performed using seven ARACNE-constructed gene networks ALK (n = 12 genes), BIRC5 (n = 45 genes), CCND1 (n = 22 genes), ERBB3 (n = 38 genes), MYCN (n = 40 genes), NTRK1 (n = 62 genes), and PHOX2B (n = 67 genes), as well as 4850 MSigDB-curated gene sets (c2, http://www.broadinstitute.org/gsea/msigdb/index.jsp, Additional file 6). The GSEA according to Subramanian *et al.*[59], was run on the “mean-per-gene” data files using the following settings: number of permutations = 1000, permutation type = gene-set, chip platform = GENE_SYMBOL.chip, enrichment statistic = weighted, metric for ranking genes = Signal2Noise, gene list sorting mode = real, gene list ordering mode = descending, max gene set size = 500 (the default), min gene set size = 10 (the default is 15). In addition, the r3 versus r1 comparisons in data sets 1–3 were investigated according to the gene list sorting mode = abs.

### Human tissue samples used for protein expression validation

Tumours histopathologically classified as GN and NB (data set 5, Table 1) were used for immunohistochemistry (4 NB and 4 GN), and immunoblot analysis (4 NB and 5 GN). Tissue from patients was obtained during surgery and stored in -80°C. Ethical approval was obtained from the Karolinska University Hospital Research Ethics Committee (Approval no. 2009/1369-31/1 and 03–736). Informed consent for using tumor samples in scientific research was provided by parents/guardians. In accordance with the approval from the Ethics Committee the informed consent was either written or verbal. When verbal or written assent was not obtained the decision was documented in the medical record.

### Immunohistochemistry

Formalin-fixed and paraffin-embedded (FFPE) tissue slides were deparaffinized in xylol and rehydrated in graded alcohols. For antigen retrieval, slides were boiled in a sodium citrate buffer (pH 6.0) for 10 min, in a microwave oven. After blocking in 1% bovine serum albumin (BSA) for 20 min, the tissue sections were incubated with primary antibody overnight, Sox10 ([N-20], Santa Cruz Biotechnology) and ErbB-3 ([RTJ2], Abcam) respectively, diluted 1:50 in 1% PBSA. Thereafter slides were rinsed in PBS and endogenous peroxidases were blocked in 0.3% H_2_O_2_ for 10 min. As a secondary antibody, anti-mouse-horseradish peroxidase (HRP) and anti-goat-horseradish peroxidase were used (Invitrogen, Paisley, UK). All slides were counterstained with haematoxylin. To control for non-specific binding, antibody specific blocking peptides and isotype-matched controls were used. For colocalization studies of Erb3 and Sox10, tumor tissue sections were simultaneously stained with primary antibodies and for fluorescence visualization, anti-goat Alexa Fluor 594 and anti-mouse Alexa Fluor 488 were used, respectively.

### Immunoblot analysis

Tumours were homogenized in RIPA buffer (20 mM Tris–HCl, pH 7.5, 150 mM NaCl, 1 mM Na_2_EDTA, 1 mM EGTA, 1% NP-40, 1% sodium deoxycholate, 2.5 mM sodium pyrophosphate, 1 mM beta-glycerophosphate, 1 mM Na_3_VO_4_, 1 ug/ml leupeptin) with protease inhibitor cocktail (Roche), 42 mM DTT and 1 mM PMSF. The total protein concentration was determined using A280 absorbance readings and 100 ug of total protein was diluted in NuPAGE® LDS sample buffer (Invitrogen) with 50 mM DTT and denatured for 10 min at 70°C. The samples were then loaded with a prestained Page Ruler protein ladder (Thermo-Scientific) on a 4-12% NuPAGE® Bis-Tris polyacrylamide gel (Invitrogen) and separated using MOPS buffer at 200V for 50 min. The proteins were transferred to PVDF membranes using NuPAGE® transfer buffer (Invitrogen) and 10% methanol. Following Ponceau staining to ensure equal loading, membranes were washed with TBS-T (Tris-buffered saline containing 0.1% Tween 20) and blocked with blocking buffer (5% milk/TBS-T) for 1 h. The primary antibodies were added to the membranes and incubated overnight at 4°C. The following day, membranes were washed with TBS-T and incubated with secondary antibodies. Following final TBS-T washes, protein detection was achieved with Pierce Super Signal® West Pico or Femto Chemiluminescent Substrate (Thermo-Scientific). The primary antibodies used were anti-ErbB3 [RTJ2] (Abcam, 1:200) and anti-Gapdh (Abcam, #ab8245, 1:10000). The secondary antibodies used were anti-mouse IgG HRP linked antibodies (Cell Signaling, #7076, 1:5000), anti-rabbit IgG HRP linked antibodies (Cell Signaling, #7074, 1:5000). All antibodies were diluted in blocking buffer.

### Statistical analyses

The expression relationship of *ERBB3* to the discriminative 6-GeneSig (*ALK*, *BIRC5*, *CCND1*, *MYCN*, *NTRK1*, and *PHOX2B*) and the ErbB family members *EGFR*, *ERBB2*, and *ERBB4* were investigated by a Pearson correlation test. The statistical significance of expression levels of *ERBB* genes (*i.e. EGFR*, *ERBB2*, *ERBB3*, and *ERBB4*) were investigated by Welch t-test. Inter-rater reliability of group assignments was tested by the Kappa statistic on crosstabs in SPSS (version 20.0).

## Competing interests

The authors declare that no competing interests exist.

## Authors' contributions

FA formulated the study design, and performed the microarray data pre-processing. AW and FA accomplished the analysis of SAM gene lists, GSEA, GO, and heat maps. AW and FA drafted the manuscript. CK performed the immunoblot analyses, and revised the manuscript. BS performed the immunohistochemistry analyses, and revised the manuscript. EK performed SAM analysis, and revised the manuscript. DD performed network modelling, and revised the manuscript. IØ performed the histopathology assessment of the Versteeg110 data set, and revised the manuscripts, KD, RS, JM, PK, and RV provided histopathology data as well as clinical data in terms of status of prognostic marker and survival of patients, and revised the manuscript. SN supervised the statistical analysis and interpretations of results, and revised the manuscript. All authors read and approved the final manuscript.

## Supplementary Material

Additional file 1 **The 6-GeneSig subgroup classification rules.** Rules based on standard deviations (SD) of expression values for all samples in each data set. In order for samples to be successfully assigned into one of four r-groups, 5 out of 6 expression rules must be met. Shaded cells indicate rules with no exception for classification into that specific subgroup. Click here for file

Additional file 2 **SAM results.** The table presents the 1000 most significant genes in each direction from the SAM analyses of four data sets: 1 (DePreter), 2 (McArdleWilzén), 3 (Wang) and 4 (Versteeg). In addition, the SAM_intersect_ gene lists are also presented (named DePreterMcArdleWilzén). Comparisons are named ′12′, ′13′, ′14′, ′23′ *etc.* corresponding to r2 versus r1, r3 versus r1, r4 versus r1, r3 versus r2, respectively. Directions of differential expressions are referred to as “up” and “down”. Click here for file

Additional file 3 **GO results.** The Biological Networks Gene Ontology tool (BiNGO) in Cytoscape was utilized to map the predominant functional themes of the SAM gene lists. The 10 most significant Gene Ontology (GO) from terms in each SAM comparison are presented. Gene lists are divided into three data sets; data set 1 & 2 (DePreterMcArdleWilzén), data set 3 (Wang), data set 4 (Versteeg), and into two differential expression directions; "up" or "down". GO-ID: Gene Ontology identification number, p-val: p-value, corr p-val: corrected p-value, Description: Description of the gene ontology theme. The "DePreterMcArdleWilzén_12_down" list was too short (22 genes) to enable the GO term search. Click here for file

Additional file 4 **Correlations of ERBB3 to the 6-GeneSig other ERBB family members.** Left panel: Pearson Correlations of *ERBB3* to the 6-gene signature (6-GeneSig) in four data sets separately (1 = De Preter, 2 = McArdle/Wilzén, 3 = Wang, 4 = Versteeg). Right panel: Pearson Correlations between the four *ERBB*-genes in four data sets separately. Positive correlations are marked in grey, and negative correlations are marked in white. Significance (2-tailed) is marked as follows: *Significant at the 0.05 level; **Significant at the 0.01 level; ***Significant at the 0.001 level. N = number of cases. Click here for file

Additional file 5 **TS candidate genes.** Tumour suppressor genes were found by the PubMed search term "Neuroblastoma AND tumour suppressor", and from previous mining of literature lists according to Vermeulen et al., Lancet Oncol. 2009 July; 10(7): 663–671 (59 gene set) and Thorell et al., BMC Med Genomics 2009 Aug 17; 2:53. Present in SAM_intersect_: genes found in the intersect SAM lists of data sets 1 & 2. y = yes; n = no. Click here for file

Additional file 6 **Gene networks used for GSEA analysis.** The table presents the gene network members of all c2 curate gene sets (4850 in total) from MSigDB (www.broadinstitute.org/gsea/msigdb), and seven ARACNE gene networks used in the GSEA analyses. Click here for file

Additional file 7 **GSEA results.** Results of Gene set enrichment analysis (GSEA) from each comparison and each data set are presented in each sheet. Data sets 1–3 (DePreter, McArdle/Wilzén, and Wang) comparisons are according to r-subgroups (r1-r4), and data set 4 (Versteeg) comparisons are according to histopathology groups; Ganglioneuroma (GN), Ganglioneuroblastoma (GNB), and Neuroblastoma (NB). Enrichments were run with 1000 permutations, permutation type = gene set, and gene list sorting mode = real (scoring both extremes) in descending order. Results per data set and comparison in each sheet are presented as follows: NAME = name of the gene set, SIZE = Size of the gene set, ES = enrichment score, NES = Normalized enrichment score, NOM p-val = Nominal p-value, FDR q-val = False Discovery Rate, FWER p-val = Familywise-error rate, RANK AT MAX = The position in the ranked list at which the maximum enrichment score occurred, LEADING EDGE = Displays the three statistics used to define the leading edge subset. In addition, the r3 versus r1 comparisons in data sets 1–3 were investigated and presented as gene list sorting mode = abs. Click here for file

Additional file 8 **Analyses of the NTRK1 and MYCN gene networks.** Networks for MYCN (n = 40) and NTRK1 (n = 62) were created from the Wang data set using the ARACNE software (see text for details). Differentially expressed genes of r-groups are marked by coloured nodes; red = up-regulated, green = down-regulated. Left panel: Data sets 1 and 2 (DePreter and McArdle/Wilzén) presenting the r3 vs. r1 comparison for the MYCN- (upper) and NTRK1- networks (lower). Only genes that were common in both data set 1 and 2 with fold change > 2 were included (*i.e.* SAM_intersect_ gene lists). Middle panel: Data set 3 (Wang) presenting the r3 vs. r1 comparison for the MYCN- (upper) and NTRK1- networks (lower). Genes included were those present in SAM gene list representing the 1000 most differentially expressed with fold change > 2 (ranked after significance). Right panel: Gene set enrichment analysis (GSEA) plots of the MYCN and NTRK1 networks are according to gene list sorting mode = real, sorted in descending order. NES = Normalized enrichment score, NOM p-val. = Nominal p-value, according to the GSEA results (see Additional file 7). *The NOM p-val. for the MYCN-network is presented according to gene list sorting mode = abs (see Additional file 7). Click here for file

Additional file 9 **The 7-GeneSig classification rules.** Rules based on standard deviations (SD) of expression values for all samples in each data set. In order to classify samples into one of the five subgroups, 5 out of 6 expression rules must be met. Shaded cells indicate rules with no exception for classification into that specific subgroup. Click here for file
