# Supplementary material for: ERBB3 is a marker of a ganglioneuroblastoma/ganglioneuroma-like expression profile in neuroblastic tumours
Source: Mol Cancer. 2013 Jul 8;12:70. doi: 10.1186/1476-4598-12-70 (PMC3766266; doi:10.1186/1476-4598-12-70)
Supplement: Additional file 1 — The 6-GeneSig subgroup classification rules. Rules based on standard deviations (SD) of expression values for all samples in each data set. In order for samples to be successfully assigned into one of four r-groups, 5 out of 6 expression rules must be met. Shaded cells indicate rules with no exception for classification into that specific subgroup. [file 1476-4598-12-70-S1.pdf]

Additional file 1. 6-GeneSig subgroup classification rules

**r-group**	**1**	**2**	**3**	**4**
ALK	below 0.2	-1 to 1.5	above 0.5	below -0.5
BIRC5	0 to -1	above 0	above 0	below 0
CCND1	-0.4 to 1.6	-0.4 to 1.6	-0.4 to 1.6	below -0.8
MYCN	below 0.5	below 0.5	above 0	below -0.5
NTRK1	above >0.5	0 to 1.5	below -0.5	below 0.4
PHOX2B	above -0.3	above -0.3	above -0.3	below 0

Rules based on standard deviations (SD) of expression values for all samples in each data set. In order for samples to be successfully assigned into one of four r-groups, 5 out of 6 expression rules must be met. Shaded cells indicate rules with no expeption for classification into that specific subgroup.
