# Supplementary material for: ERBB3 is a marker of a ganglioneuroblastoma/ganglioneuroma-like expression profile in neuroblastic tumours
Source: Mol Cancer. 2013 Jul 8;12:70. doi: 10.1186/1476-4598-12-70 (PMC3766266; doi:10.1186/1476-4598-12-70)
Supplement: Additional file 3 — GO results. The Biological Networks Gene Ontology tool (BiNGO) in Cytoscape was utilized to map the predominant functional themes of the SAM gene lists. The 10 most significant Gene Ontology (GO) from terms in each SAM comparison are presented. Gene lists are divided into three data sets; data set 1 & 2 (DePreterMcArdleWilzén), data set 3 (Wang), data set 4 (Versteeg), and into two differential expression directions; "up" or "down". GO-ID: Gene Ontology identification number, p-val: p-value, corr p-val: corrected p-value, Description: Description of the gene ontology theme. The "DePreterMcArdleWilzén_12_down" list was too short (22 genes) to enable the GO term search. [file 1476-4598-12-70-S3.pdf]

Additional file 3. GO search results

DePreterMcArdleWilzén_12_up

GO-ID	p-value	corr p-value	Description
7049	1.8112E-35	1.2213E-32	cell cycle
279	2.2046E-35	1.2213E-32	M phase
22403	1.1612E-34	4.2886E-32	cell cycle phase
22402	2.4998E-32	6.9245E-30	cell cycle process
280	3.8753E-31	7.1564E-29	nuclear division
7067	3.8753E-31	7.1564E-29	mitosis
87	9.7333E-31	1.5406E-28	M phase of mitotic cell cycle
48285	1.2594E-30	1.7443E-28	organelle fission
278	6.4166E-30	7.8995E-28	mitotic cell cycle
51301	4.0184E-27	4.4523E-25	cell division

DePreterMcArdleWilzén_12_down

Not available

DePreterMcArdleWilzén_13_up

GO-ID	p-value	corr p-value	Description
7049	1.7440E-31	3.1549E-28	cell cycle
22403	4.5553E-30	4.1203E-27	cell cycle phase
278	1.1321E-29	6.8266E-27	mitotic cell cycle
280	1.4262E-28	5.1599E-26	nuclear division
7067	1.4262E-28	5.1599E-26	mitosis
87	4.4675E-28	1.3470E-25	M phase of mitotic cell cycle
279	5.2160E-28	1.3480E-25	M phase
48285	6.1464E-28	1.3899E-25	organelle fission
22402	8.1476E-28	1.6377E-25	cell cycle process
34641	2.0224E-22	3.6585E-20	cellular nitrogen compound metabolic process

DePreterMcArdleWilzén_13_down

GO-ID	p-value	corr p-value	Description
51179	3.4144E-10	6.2040E-7	localization
7399	2.5524E-9	2.3189E-6	nervous system development
7611	1.5149E-8	8.4301E-6	learning or memory
23052	2.1684E-8	8.4301E-6	signaling
51234	2.8496E-8	8.4301E-6	establishment of localization
23060	3.2477E-8	8.4301E-6	signal transmission
23046	3.2477E-8	8.4301E-6	signaling process
6810	3.8549E-8	8.7554E-6	transport
6836	5.2482E-7	1.0596E-4	neurotransmitter transport
7613	3.8893E-6	7.0668E-4	memory

DePreterMcArdleWilzén_14_up

GO-ID	p-value	corr p-value	Description
48731	1.9103E-12	4.1168E-9	system development
48856	1.4247E-11	1.5352E-8	anatomical structure development
7275	2.2730E-11	1.6328E-8	multicellular organismal development
22008	4.2232E-11	1.9176E-8	neurogenesis
9611	4.4492E-11	1.9176E-8	response to wounding
32502	1.0767E-9	3.7074E-7	developmental process
48518	1.2043E-9	3.7074E-7	positive regulation of biological process
7399	2.5337E-9	6.8253E-7	nervous system development
32501	7.1736E-9	1.7177E-6	multicellular organismal process
6956	1.1442E-8	2.4420E-6	complement activation

DePreterMcArdleWilzén_14_down

GO-ID	p-value	corr p-value	Description
7399	3.7004E-7	6.0761E-4	nervous system development
9394	1.1724E-5	9.5552E-3	2'-deoxyribonucleotide metabolic process
42423	2.1576E-5	9.5552E-3	catecholamine biosynthetic process
1764	2.3277E-5	9.5552E-3	neuron migration
9262	4.3249E-5	1.4203E-2	deoxyribonucleotide metabolic process
48699	7.6800E-5	1.6888E-2	generation of neurons
22008	7.7181E-5	1.6888E-2	neurogenesis
7275	8.2280E-5	1.6888E-2	multicellular organismal development
32502	1.1320E-4	2.0652E-2	developmental process
34641	1.6387E-4	2.6907E-2	cellular nitrogen compound metabolic process

DePreterMcArdleWilzén_23_up

GO-ID	p-value	corr p-value	Description
44271	1.6978E-5	1.2718E-2	cellular nitrogen compound biosynthetic process
46112	3.8543E-5	1.2718E-2	nucleobase biosynthetic process
6520	5.0853E-5	1.2718E-2	cellular amino acid metabolic process
51656	5.6811E-5	1.2718E-2	establishment of organelle localization
44106	6.7264E-5	1.2718E-2	cellular amine metabolic process
6189	9.7126E-5	1.2718E-2	'de novo' IMP biosynthetic process
48846	9.7126E-5	1.2718E-2	axon extension involved in axon guidance
6541	1.1669E-4	1.2718E-2	glutamine metabolic process
9168	1.1669E-4	1.2718E-2	purine ribonucleoside monophosphate biosynthetic process
9127	1.1669E-4	1.2718E-2	purine nucleoside monophosphate biosynthetic process

DePreterMcArdleWilzén_23_down

GO-ID	p-value	corr p-value	Description
23052	2.6014E-8	4.1129E-5	signaling
51179	8.0624E-8	6.3733E-5	localization
23060	4.5728E-6	1.8074E-3	signal transmission
23046	4.5728E-6	1.8074E-3	signaling process
6810	6.7623E-6	2.1382E-3	transport
51234	9.7585E-6	2.5714E-3	establishment of localization
32502	1.6497E-5	3.7260E-3	developmental process
16265	2.4730E-5	3.9237E-3	death
7399	2.9299E-5	3.9237E-3	nervous system development
7275	3.1726E-5	3.9237E-3	multicellular organismal development

DePreterMcArdleWilzén_24_up

GO-ID	p-value	corr p-value	Description
48731	5.7794E-13	1.2356E-9	system development
48856	3.5341E-12	2.7983E-9	anatomical structure development
7275	3.9265E-12	2.7983E-9	multicellular organismal development
7399	1.7395E-11	9.2978E-9	nervous system development
32502	1.8137E-10	7.7554E-8	developmental process
22008	4.1526E-10	1.4797E-7	neurogenesis
50793	2.1690E-9	6.6248E-7	regulation of developmental process
48518	1.1481E-8	3.0684E-6	positive regulation of biological process
32501	1.2990E-8	3.0858E-6	multicellular organismal process
48699	1.9981E-8	4.2720E-6	generation of neurons

DePreterMcArdleWilzén_24_down

GO-ID	p-value	corr p-value	Description
22403	6.1947E-20	6.3442E-17	cell cycle phase
7049	7.1244E-20	6.3442E-17	cell cycle
278	1.2891E-18	7.6531E-16	mitotic cell cycle
279	5.3884E-18	1.7419E-15	M phase
280	5.8684E-18	1.7419E-15	nuclear division
7067	5.8684E-18	1.7419E-15	mitosis
87	1.3733E-17	3.4459E-15	M phase of mitotic cell cycle
22402	1.6534E-17	3.4459E-15	cell cycle process
48285	1.7413E-17	3.4459E-15	organelle fission
51301	5.7542E-16	1.0248E-13	cell division

DePreterMcArdleWilzén_34_up

GO-ID	p-value	corr p-value	Description
48731	2.0111E-12	4.4909E-9	system development
7275	7.8278E-12	8.7398E-9	multicellular organismal development
48856	2.0158E-11	1.5004E-8	anatomical structure development
32502	5.1079E-10	2.8515E-7	developmental process
32501	2.8380E-9	1.2675E-6	multicellular organismal process
9611	2.4557E-8	9.1392E-6	response to wounding
23052	5.3646E-8	1.7113E-5	signaling
6959	9.4924E-8	2.6496E-5	humoral immune response
6956	3.8381E-7	9.5226E-5	complement activation
2541	4.6967E-7	9.9155E-5	activation of plasma proteins involved in acute inflammatory response

DePreterMcArdleWilzén_34_down

GO-ID	p-value	corr p-value	Description
6139	9.4150E-36	1.8030E-32	nucleobase, nucleoside, nucleotide and nucleic acid metabolic process
90304	1.7313E-33	1.6577E-30	nucleic acid metabolic process
34641	2.6155E-33	1.6696E-30	cellular nitrogen compound metabolic process
6807	2.4146E-31	1.1560E-28	nitrogen compound metabolic process
44260	9.7791E-24	3.7454E-21	cellular macromolecule metabolic process
6260	5.2066E-21	1.6618E-18	DNA replication
6259	3.8638E-20	1.0319E-17	DNA metabolic process
7049	4.3110E-20	1.0319E-17	cell cycle
44237	9.3551E-20	1.9906E-17	cellular metabolic process
43170	1.9474E-19	3.7292E-17	macromolecule metabolic process

Wang_12_up

GO-ID	p-value	corr p-value	Description
6334	1.0645E-39	3.0371E-36	nucleosome assembly
65004	4.6799E-39	6.6758E-36	protein-DNA complex assembly
31497	8.5573E-39	8.1380E-36	chromatin assembly
6323	6.8053E-38	4.8539E-35	DNA packaging
34728	1.6113E-37	9.1942E-35	nucleosome organization
71103	5.1994E-37	2.4723E-34	DNA conformation change
6333	5.3465E-34	2.1791E-31	chromatin assembly or disassembly
34622	6.1973E-32	2.2101E-29	cellular macromolecular complex assembly
34621	1.7800E-31	5.6425E-29	cellular macromolecular complex subunit organization
9987	3.2750E-31	9.3434E-29	cellular process

Wang_12_down

GO-ID	p-value	corr p-value	Description
7156	1.8517E-13	4.5847E-10	homophilic cell adhesion
9987	3.0998E-8	3.8375E-5	cellular process
16337	1.6178E-7	1.3352E-4	cell-cell adhesion
48523	5.7651E-7	3.3702E-4	negative regulation of cellular process
6464	8.0529E-7	3.3702E-4	protein modification process
45941	9.3830E-7	3.3702E-4	positive regulation of transcription
42147	1.0801E-6	3.3702E-4	retrograde transport, endosome to Golgi
43687	1.0889E-6	3.3702E-4	post-translational protein modification
10628	3.1218E-6	8.5883E-4	positive regulation of gene expression
45935	4.5382E-6	1.0360E-3	positive regulation of nucleobase, nucleoside, nucleotide and nucleic acid metabolic process

Wang_13_up

GO-ID	p-value	corr p-value	Description
44237	1.1533E-40	2.8129E-37	cellular metabolic process
6139	1.6662E-38	2.0320E-35	nucleobase, nucleoside, nucleotide and nucleic acid metabolic process
44260	7.8058E-37	6.3461E-34	cellular macromolecule metabolic process
34641	1.0993E-35	5.8270E-33	cellular nitrogen compound metabolic process
90304	1.1946E-35	5.8270E-33	nucleic acid metabolic process
6807	1.2493E-33	5.0782E-31	nitrogen compound metabolic process
44238	3.6841E-31	1.2837E-28	primary metabolic process
34645	2.4281E-30	7.4025E-28	cellular macromolecule biosynthetic process
9059	1.4895E-29	4.0364E-27	macromolecule biosynthetic process
44249	2.6682E-29	6.5077E-27	cellular biosynthetic process

Wang_13_down

GO-ID	p-value	corr p-value	Description
7156	1.7530E-21	5.0206E-18	homophilic cell adhesion
16337	1.0892E-16	1.5597E-13	cell-cell adhesion
19882	2.0881E-15	1.9935E-12	antigen processing and presentation
2504	2.3547E-14	1.6860E-11	antigen processing and presentation of peptide or polysaccharide antigen via MHC class II
23052	2.1435E-12	1.2278E-9	signaling
23060	2.9191E-11	1.1943E-8	signal transmission
23046	2.9191E-11	1.1943E-8	signaling process
7155	1.6473E-10	5.5369E-8	cell adhesion
22610	1.7399E-10	5.5369E-8	biological adhesion
7165	1.2405E-8	3.5528E-6	signal transduction

Wang_14_up

GO-ID	p-value	corr p-value	Description
9611	1.1549E-15	3.6160E-12	response to wounding
6950	6.0543E-15	9.4781E-12	response to stress
2376	2.1914E-12	2.0662E-9	immune system process
50896	2.6396E-12	2.0662E-9	response to stimulus
42060	1.5046E-11	9.4215E-9	wound healing
22603	1.9016E-11	9.9234E-9	regulation of anatomical structure morphogenesis
51239	3.2850E-11	1.4693E-8	regulation of multicellular organismal process
48518	4.6879E-11	1.8347E-8	positive regulation of biological process
48646	5.5512E-11	1.9312E-8	anatomical structure formation involved in morphogenesis
23052	7.9009E-11	2.4738E-8	signaling

Wang_14_down

GO-ID	p-value	corr p-value	Description
60255	5.4606E-25	6.7948E-22	regulation of macromolecule metabolic process
10468	5.7413E-25	6.7948E-22	regulation of gene expression
10556	1.7295E-24	1.3646E-21	regulation of macromolecule biosynthetic process
45449	4.9484E-24	2.9282E-21	regulation of transcription
31326	8.8935E-24	4.2102E-21	regulation of cellular biosynthetic process
31323	1.2481E-23	4.9238E-21	regulation of cellular metabolic process
51276	1.7285E-23	5.4137E-21	chromosome organization
19219	1.9139E-23	5.4137E-21	regulation of nucleobase, nucleoside, nucleotide and nucleic acid metabolic process
19222	2.0585E-23	5.4137E-21	regulation of metabolic process
9889	2.3096E-23	5.4669E-21	regulation of biosynthetic process

Wang_23_up

GO-ID	p-value	corr p-value	Description
10467	6.6449E-36	1.6373E-32	gene expression
6139	4.4919E-31	5.5341E-28	nucleobase, nucleoside, nucleotide and nucleic acid metabolic process
44237	8.9845E-31	7.1159E-28	cellular metabolic process
34641	1.1552E-30	7.1159E-28	cellular nitrogen compound metabolic process
6807	2.1200E-29	1.0447E-26	nitrogen compound metabolic process
6412	1.2095E-27	4.9672E-25	translation
44260	2.2270E-26	7.8390E-24	cellular macromolecule metabolic process
6414	9.3140E-26	2.8687E-23	translational elongation
44238	1.1535E-25	3.1581E-23	primary metabolic process
90304	7.1899E-25	1.7716E-22	nucleic acid metabolic process

Wang_23_down

GO-ID	p-value	corr p-value	Description
19882	1.3205E-14	1.8988E-11	antigen processing and presentation
2504	1.3376E-14	1.8988E-11	antigen processing and presentation of peptide or polysaccharide antigen via MHC class II
23052	6.3149E-11	5.9760E-8	signaling
48731	5.1542E-10	3.6582E-7	system development
48856	7.1174E-9	3.6886E-6	anatomical structure development
6955	7.8484E-9	3.6886E-6	immune response
9987	9.0948E-9	3.6886E-6	cellular process
23060	2.8623E-8	8.9970E-6	signal transmission
23046	2.8623E-8	8.9970E-6	signaling process
7399	3.3881E-8	8.9970E-6	nervous system development

Wang_24_up

GO-ID	p-value	corr p-value	Description
48518	7.4800E-15	2.3053E-11	positive regulation of biological process
9611	2.9007E-13	3.2089E-10	response to wounding
23052	3.1235E-13	3.2089E-10	signaling
42221	6.2468E-13	4.8131E-10	response to chemical stimulus
10033	1.6211E-12	9.9925E-10	response to organic substance
2376	3.1964E-11	1.5976E-8	immune system process
48731	3.8947E-11	1.5976E-8	system development
50793	4.3898E-11	1.5976E-8	regulation of developmental process
48522	4.6652E-11	1.5976E-8	positive regulation of cellular process
65007	5.5263E-11	1.7032E-8	biological regulation

Wang_24_down

GO-ID	p-value	corr p-value	Description
90304	3.7432E-35	8.2237E-32	nucleic acid metabolic process
51276	4.6709E-34	5.1310E-31	chromosome organization
7049	1.4280E-33	1.0458E-30	cell cycle
22403	2.9992E-33	1.6473E-30	cell cycle phase
6996	7.3401E-33	3.2253E-30	organelle organization
6259	2.7661E-31	1.0129E-28	DNA metabolic process
278	3.3646E-30	1.0560E-27	mitotic cell cycle
6139	4.2957E-30	1.1797E-27	nucleobase, nucleoside, nucleotide and nucleic acid metabolic process
279	1.1182E-29	2.7297E-27	M phase
22402	1.5275E-28	3.3559E-26	cell cycle process

Wang_34_up

GO-ID	p-value	corr p-value	Description
2376	8.2047E-21	2.4795E-17	immune system process
19882	2.7820E-19	4.2035E-16	antigen processing and presentation
6955	4.7667E-19	4.8017E-16	immune response
50896	3.7900E-17	2.8634E-14	response to stimulus
2504	1.0062E-12	6.0816E-10	antigen processing and presentation of peptide or polysaccharide antigen via MHC class II
23052	1.7885E-12	9.0083E-10	signaling
9611	7.0776E-12	2.7651E-9	response to wounding
6950	7.3201E-12	2.7651E-9	response to stress
48518	8.8506E-12	2.9718E-9	positive regulation of biological process
42221	2.8293E-11	8.5500E-9	response to chemical stimulus

Wang_34_down

GO-ID	p-value	corr p-value	Description
90304	2.2727E-64	4.9818E-61	nucleic acid metabolic process
6139	1.7034E-63	1.8670E-60	nucleobase, nucleoside, nucleotide and nucleic acid metabolic process
34641	5.6746E-55	4.1463E-52	cellular nitrogen compound metabolic process
44260	3.0141E-54	1.6517E-51	cellular macromolecule metabolic process
6807	7.0009E-51	3.0692E-48	nitrogen compound metabolic process
43170	5.1546E-43	1.8831E-40	macromolecule metabolic process
6259	2.8942E-40	9.0629E-38	DNA metabolic process
44237	1.5683E-39	4.2971E-37	cellular metabolic process
22403	1.7314E-37	4.2170E-35	cell cycle phase
10467	1.2676E-36	2.7786E-34	gene expression

Versteeg_NB-GN_up

GO-ID	p-value	corr p-value	Description
23052	2.0379E-12	5.4454E-9	signaling
23060	2.8975E-10	2.5807E-7	signal transmission
23046	2.8975E-10	2.5807E-7	signaling process
48731	8.8133E-10	5.8437E-7	system development
7399	1.0935E-9	5.8437E-7	nervous system development
7275	2.6000E-9	1.1579E-6	multicellular organismal development
48856	3.6539E-9	1.3947E-6	anatomical structure development
51239	4.4860E-9	1.4983E-6	regulation of multicellular organismal process
50793	2.0626E-8	6.1235E-6	regulation of developmental process
32502	2.6964E-8	7.2048E-6	developmental process

Versteeg_NB-GN_down

GO-ID	p-value	corr p-value	Description
279	1.0202E-40	2.0128E-37	M phase
22403	1.6713E-39	1.6487E-36	cell cycle phase
87	8.8385E-37	5.8128E-34	M phase of mitotic cell cycle
51301	1.9552E-36	9.6440E-34	cell division
7049	5.4729E-36	2.1596E-33	cell cycle
280	3.1111E-35	8.7690E-33	nuclear division
7067	3.1111E-35	8.7690E-33	mitosis
22402	2.3357E-34	5.2401E-32	cell cycle process
48285	2.3903E-34	5.2401E-32	organelle fission
278	1.4824E-33	2.9249E-31	mitotic cell cycle

Versteeg_NB-GNB_up

GO-ID	p-value	corr p-value	Description
7267	4.3871E-6	1.0871E-2	cell-cell signaling
7154	1.1659E-5	1.3090E-2	cell communication
90032	2.2458E-5	1.3090E-2	negative regulation of steroid hormone biosynthetic process
7268	2.4302E-5	1.3090E-2	synaptic transmission
44057	2.9470E-5	1.3090E-2	regulation of system process
23052	3.1696E-5	1.3090E-2	signaling
51969	3.9451E-5	1.3966E-2	regulation of transmission of nerve impulse
47496	7.0953E-5	1.6762E-2	vesicle transport along microtubule
50804	7.3703E-5	1.6762E-2	regulation of synaptic transmission
30641	8.2456E-5	1.6762E-2	regulation of cellular pH

Versteeg_NB-GNB_down

GO-ID	p-value	corr p-value	Description
279	5.3155E-41	9.9878E-38	M phase
22403	2.2877E-40	2.1493E-37	cell cycle phase
51301	4.3424E-39	2.6917E-36	cell division
87	5.7301E-39	2.6917E-36	M phase of mitotic cell cycle
280	1.7270E-37	5.4085E-35	nuclear division
7067	1.7270E-37	5.4085E-35	mitosis
7049	3.8771E-37	1.0407E-34	cell cycle
278	9.8637E-37	2.3167E-34	mitotic cell cycle
48285	1.5826E-36	3.3040E-34	organelle fission
22402	1.0873E-34	2.0431E-32	cell cycle process

Versteeg_GNB-GN_up

GO-ID	p-value	corr p-value	Description
10627	1.0329E-7	2.7280E-4	regulation of intracellular protein kinase cascade
23033	9.7516E-7	7.9084E-4	signaling pathway
7165	1.1828E-6	7.9084E-4	signal transduction
35556	1.1978E-6	7.9084E-4	intracellular signal transduction
23052	2.2404E-6	1.0688E-3	signaling
23060	2.9144E-6	1.0688E-3	signal transmission
23046	2.9144E-6	1.0688E-3	signaling process
23014	3.6422E-6	1.0688E-3	signal transmission via phosphorylation event
7243	3.6422E-6	1.0688E-3	intracellular protein kinase cascade
7259	4.9701E-6	1.3126E-3	JAK-STAT cascade

Versteeg_GNB-GN_down

GO-ID	p-value	corr p-value	Description
7268	3.1378E-8	6.7808E-5	synaptic transmission
19226	7.3869E-8	7.9815E-5	transmission of nerve impulse
1505	1.4180E-7	1.0214E-4	regulation of neurotransmitter levels
7611	1.2044E-6	5.7590E-4	learning or memory
6836	1.3325E-6	5.7590E-4	neurotransmitter transport
6810	2.3340E-6	8.4062E-4	transport
51234	4.2605E-6	1.3153E-3	establishment of localization
7612	1.1774E-5	2.9652E-3	learning
51179	1.3631E-5	2.9652E-3	localization
48489	1.3721E-5	2.9652E-3	synaptic vesicle transport

The Biological Networks Gene Ontology tool (BiNGO) in Cytoscape was utilized to map the predominant functional themes of the SAM gene lists. The 10 most significant Gene Ontology (GO) from terms in each SAM comparison are presented. Gene lists are divided into three data sets; data set 1 & 2 (DePreterMcArdleWilzén), data set 3 (Wang), data set 4 (Versteeg), and into two differential expression directions; "up" or "down". GO-ID: Gene Ontology identification number, p-val: p-value, corr p-val: corrected p-value, Description: Description of the gene ontology theme. The "DePreterMcArdleWilzén_12_down" list was too short (22 genes) to enable the GO term search.
