# Supplementary material for: ERBB3 is a marker of a ganglioneuroblastoma/ganglioneuroma-like expression profile in neuroblastic tumours
Source: Mol Cancer. 2013 Jul 8;12:70. doi: 10.1186/1476-4598-12-70 (PMC3766266; doi:10.1186/1476-4598-12-70)
Supplement: Additional file 4 — Correlations of ERBB3 to the 6-GeneSig other ERBB family members. Left panel: Pearson Correlations of ERBB3 to the 6-gene signature (6-GeneSig) in four data sets separately (1 = De Preter, 2 = McArdle/Wilzén, 3 = Wang, 4 = Versteeg). Right panel: Pearson Correlations between the four ERBB-genes in four data sets separately. Positive correlations are marked in grey, and negative correlations are marked in white. Significance (2-tailed) is marked as follows: *Significant at the 0.05 level; **Significant at the 0.01 level; ***Significant at the 0.001 level. N = number of cases. [file 1476-4598-12-70-S4.pdf]

Additional file 4. Correlations of *ERBB3* to the 6-GeneSig and other *ERBB* family members

	Data set	*ERBB3* 1	*ERBB3* 2	*ERBB3* 3	*ERBB3* 4
ALK	PC	-0.201	-0.343	-0.279	-0.377
	Sign			**	***
	N	17	30	102	110
BIRC5	PC	-0.451	-0.486	-0.452	-0.626
	Sign		**	**	***
	N	17	30	102	110
CCND1	PC	-0.305	-0.599	-0.253	-0.455
	Sign		***	*	**
	N	17	30	102	110
MYCN	PC	-0.421	-0.454	-0.290	-0.551
	Sign		*	**	***
	N	17	30	102	110
NTRK1	PC	-0.063	-0.223	0.266	-0.135
	Sign			**	
	N	17	30	102	110
PHOX2B	PC	-0.803	-0.782	-0.216	-0.739
	Sign	***	***	*	***
	N	17	30	102	110

		EGFR				***ERBB2***				***ERBB3***			
	**Data set**	1	2	3	4	1	2	3	4	1	2	3	4
ERBB4	PC	0.236	-0.14	0.124	-0.056	-0.114	-0.154	-0.107	-0.17	-0.058	-0.206	0.059	-0.219
	Sign												*
	N	17	30	102	110	17	30	102	110	17	30	102	110
EGFR	PC					0.411	0.705	0.250	0.822	0.4	0.819	0.359	0.769
	Sign						***	*	***		***	***	***
	N					17	30	102	110	17	30	102	110
ERBB2	PC									0.623	0.881	0.414	0.800
	Sign									***	***	***	***
	N									17	30	102	110

Left panel: Pearson Correlations (PC) of *ERBB3* to the 6-gene signature (6-GeneSig) in four data sets separately (1= De Preter, 2= McArdle/Wilzén, 3= Wang, 4=Versteeg). Right panel: Pearson Correlations between the four *ERBB*-genes in four data sets separately. Positive correlations are marked in grey, and negative correlations are marked in white. Significance (Sign; 2-tailed) is marked as follows: *Significant at the 0.05 level; **Significant at the 0.01 level; ***Significant at the 0.001 level. N=number of cases.
