# Supplementary material for: ERBB3 is a marker of a ganglioneuroblastoma/ganglioneuroma-like expression profile in neuroblastic tumours
Source: Mol Cancer. 2013 Jul 8;12:70. doi: 10.1186/1476-4598-12-70 (PMC3766266; doi:10.1186/1476-4598-12-70)
Supplement: Additional file 5 — TS candidate genes. Tumour suppressor genes were found by the PubMed search term "Neuroblastoma AND tumour suppressor", and from previous mining of literature lists according to Vermeulen et al., Lancet Oncol. 2009 July; 10(7): 663–671 (59 gene set) and Thorell et al., BMC Med Genomics 2009 Aug 17; 2:53. Present in SAMintersect: genes found in the intersect SAM lists of data sets 1 & 2. y = yes; n = no. [file 1476-4598-12-70-S5.pdf]

Additional file 5. TS candidate genes

Gene name	Selected by	Present in $SAM_{intersect}$	Localization	References (Search query "neuroblastoma AND tumour suppressor")
CACNA2D3	Thorell et al., 2009	y	3p21.1	Thorell K et al., BMC Med Genomics. 2009 Aug 17;2:53.
CAMTA1	Vermeulen et al., 2009	y	1p36.31-26.23	Henrich KO et al., Cancer Res. 2011 Apr 15;71(8):3142-51.
CD44	Vermeulen et al., 2009	y	11p13	Hoebeeck J et al., Cancer Lett. 2009 Jan 18;273(2):336-46. Taran K et al., Folia Neuropathol. 2007;45(3):126-32.
CDC42	Location	y	1p36.1	Valentijn LJ et al., Cancer Res. 2005 Apr 15;65(8):3136-45.
CHD5	Vermeulen et al., 2009	y	1p36.31	White PS et al., Oncogene. 2005 Apr 14;24(16):2684-94. Garcia I et al., Mol Cancer. 2010 Oct 15;9:277. Koyama H et al., Clin Cancer Res. 2012 Mar 15;18(6):1588-97.
CLSTN1	Vermeulen et al., 2009	y	1p36.22	Fransson S et al., Genes Chromosomes Cancer. 2007 Jan;46(1):45-52. Janoueix-Lerosey I et al., Oncogene. 2004 Aug 5;23(35):5912-22.
CNTNAP2	Thorell et al., 2009	y	7p35	Thorell K et al., BMC Med Genomics. 2009 Aug 17;2:53.
CTNNBIP1	Location	y	1p36.2	Fransson S et al., Genes Chromosomes Cancer. 2007 Jan;46(1):45-52.
DKK3	Literature	y	11p15.3	De Brouwer S et al., Int J Cancer. 2012 Jun 1;130(11):2591-8. Haug BH et al., Carcinogenesis. 2011 Jul;32(7):1005-12.
KIF1B	Location	y	1p36.2	Munirajan AK et al., J Biol Chem. 2008 Sep 5;283(36):24426-34. Carén H et al., Mol Cancer. 2005 Mar 1;4(1):10. Krona C et al., Oncogene. 2003 Apr 17;22(15):2343-51. Ochiai H et al., Oncogene. 2010 May 6;29(18):2681-90. Ohira M et al., Oncogene. 2000 Aug 31;19(37):4302-7.
PIK3CD	Location	y	1p36.22	Carén H et al., Br J Cancer. 2007 Nov 19;97(10):1416-24.
PRDM2 (alias RIZ)	Vermeulen et al., 2009	y	1p36.2	Geli J et al., Int J Oncol. 2010 Nov;37(5):1323-30. Hoebeeck J et al., Cancer Lett. 2009 Jan 18;273(2):336-46.
PTN	Vermeulen et al., 2009	y	7q33	Nakagawara A et al., Cancer Res. 1995 Apr 15;55(8):1792-7.
SEMA3B	Thorell et al., 2009	y	3p21.31	Nair PN et al., Cancer Genet Cytogenet. 2007 Apr 15;174(2):100-10.
TFAP2B	Thorell et al., 2009	y	6p12.3	Thorell K et al., BMC Med Genomics. 2009 Aug 17;2:53.
CADM1 (alias IGSF4 & TSLC1)	Vermeulen et al., 2009	n	11q23.3	Michels E et al., BMC Cancer. 2008 Jun 17;8:173. Ando K et al., Int J Cancer. 2008 Nov 1;123(9):2087-94.
CASP8	Literature (methylation)	n	2q33.1	Hoebeeck J et al., Cancer Lett. 2009 Jan 18;273(2):336-46.
CASZ1	Location	n	1p36.22	Liu Z et al., Cell Death Differ. 2011 Jul;18(7):1174-83. Carén H et al., Br J Cancer. 2007 Nov 19;97(10):1416-24.
CDH1	Literature (methylation)	n	16q22.1	Hoebeeck J et al., Cancer Lett. 2009 Jan 18;273(2):336-46.
ENO1	Location	n	1p36.2	Ejeskär K et al., BMC Cancer. 2005 Dec 16;5:161.
GNB1	Vermeulen et al., 2009	n	1p36.33	Thorell K et al., BMC Med Genomics. 2009 Aug 17;2:53. Janoueix-Lerosey I et al., Oncogene. 2004 Aug 5;23(35):5912-22.
KRT19	Literature (methylation)	n	17q21.2	Carén H et al., BMC Cancer. 2011 Feb 11;11:66.
POU4F2	Thorell et al., 2009	n	4q31.2	Thorell K et al., BMC Med Genomics. 2009 Aug 17;2:53.
PTEN	Literature (methylation)	n	10q23.31	Hoebeeck J et al., Cancer Lett. 2009 Jan 18;273(2):336-46.
PTPRD	Literature	n	9p24.1	Clark O et al., Cancer Invest. 2012 Jun;30(5):422-32. Meehan M et al., Mol Cancer. 2012 Feb 5;11:6. Stallings RL et al., Cancer Res. 2006 Apr 1;66(7):3673-80.
RASSF1	Literature (methylation)	n	3p21.31	Hoebeeck J et al., Cancer Lett. 2009 Jan 18;273(2):336-46. Michalowski MB et al., Pediatr Blood Cancer. 2008 Jan;50(1):29-32.
RASSF5 (alias NORE1A)	Literature (methylation)	n	1q32.1	Djos A et al., Mol Cancer. 2012 Jun 13;11(1):40. Geli J et al., Int J Cancer. 2008 Jul 15;123(2):389-94.
RASSF6	Literature (methylation)	n	4q13.3	Djos A et al., Mol Cancer. 2012 Jun 13;11(1):40.
RASSF7	Literature (methylation)	n	11p15.5	Djos A et al., Mol Cancer. 2012 Jun 13;11(1):40.
SLC35E2	Thorell et al., 2009	n	1p36.33	Thorell K et al., BMC Med Genomics. 2009 Aug 17;2:53.
TNFRSF25 (alias APO3)	Vermeulen et al., 2009	n	1p36.2	Eggert A et al., Eur J Cancer. 2002 Jan;38(1):92-8. Okawa ER et al., Oncogene. 2008 Jan 31;27(6):803-10.
UBE4B	Location	n	1p36.2	Krona C et al., Oncogene. 2003 Apr 17;22(15):2343-51.
ZMYND10	Literature (methylation)	n	3p21.31	Hoebeeck J et al., Cancer Lett. 2009 Jan 18;273(2):336-46.

Tumour suppressor genes were found by the PubMed search term "Neuroblastoma AND tumour suppressor", and from previous mining of literature lists according to Vermeulen et al., Lancet Oncol. 2009 July; 10(7): 663–671 (59 gene set) and Thorell et al., BMC Med Genomics 2009 Aug 17;2:53. Present in $SAM_{intersect:}$ genes found in the intersect SAM lists of data sets 1 & 2. y=yes; n=no.
