# Supplementary figures and images for: ERBB3 is a marker of a ganglioneuroblastoma/ganglioneuroma-like expression profile in neuroblastic tumours
Source: Mol Cancer. 2013 Jul 8;12:70. doi: 10.1186/1476-4598-12-70 (PMC3766266; doi:10.1186/1476-4598-12-70)

### Data set 3: r3 vs r1

[illegible]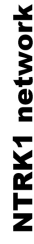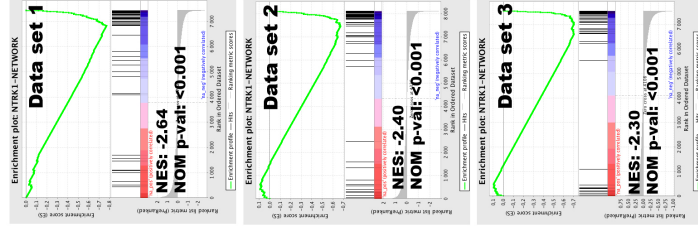

Supplement: Additional file 8 — Analyses of the NTRK1 and MYCN gene networks. Networks for MYCN (n = 40) and NTRK1 (n = 62) were created from the Wang data set using the ARACNE software (see text for details). Differentially expressed genes of r-groups are marked by coloured nodes; red = up-regulated, green = down-regulated. Left panel: Data sets 1 and 2 (DePreter and McArdle/Wilzén) presenting the r3 vs. r1 comparison for the MYCN- (upper) and NTRK1- networks (lower). Only genes that were common in both data set 1 and 2 with fold change > 2 were included (i.e. SAMintersect gene lists). Middle panel: Data set 3 (Wang) presenting the r3 vs. r1 comparison for the MYCN- (upper) and NTRK1- networks (lower). Genes included were those present in SAM gene list representing the 1000 most differentially expressed with fold change > 2 (ranked after significance). Right panel: Gene set enrichment analysis (GSEA) plots of the MYCN and NTRK1 networks are according to gene list sorting mode = real, sorted in descending order. NES = Normalized enrichment score, NOM p-val. = Nominal p-value, according to the GSEA results (see Additional file 7). *The NOM p-val. for the MYCN-network is presented according to gene list sorting mode = abs (see Additional file 7). [file 1476-4598-12-70-S8.pdf]
