# Supplementary material for: ERBB3 is a marker of a ganglioneuroblastoma/ganglioneuroma-like expression profile in neuroblastic tumours
Source: Mol Cancer. 2013 Jul 8;12:70. doi: 10.1186/1476-4598-12-70 (PMC3766266; doi:10.1186/1476-4598-12-70)
Supplement: Additional file 9 — The 7-GeneSig classification rules. Rules based on standard deviations (SD) of expression values for all samples in each data set. In order to classify samples into one of the five subgroups, 5 out of 6 expression rules must be met. Shaded cells indicate rules with no exception for classification into that specific subgroup. [file 1476-4598-12-70-S9.pdf]

Additional file 9. The 7-GeneSig classification rules

| Group | NB-r1 | NB-r2 | NB-r3 | GN-r4 | GNB-r4 |
|---|---|---|---|---|---|
| ALK | below 0.2 | -1 to 1.5 | above 0.5 | below -0.5 | -0.5 to 0.5 |
| BIRC5 | 0 to -1 | above 0 | above 0 | below 0 | below 0 |
| CCND1 | -0.4 to 1.6 | -0.4 to 1.6 | -0.4 to 1.6 | below -0.8 | below 0 |
| MYCN | below 0.5 | below 0.5 | above 0 | below -0.5 | below 0 |
| NTRK1 | above >0.5 | 0 to 1.5 | below -0.5 | below 0.4 | below 1 |
| PHOX2B | above -0.3 | above -0.3 | above -0.3 | below -1.5 | -1.5 to 0 |
| ERBB3 | below 1 | below 0 | below 0 | above 1 | -1 to 1 |

Rules based on standard deviations (SD) of expression values for all samples in each data set. In order to classify samples into one of the five subgroups, 5 out of 6 expression rules must be met. Shaded cells indicate rules with no exception for classification into that specific subgroup.
